# A Systematic Review of Randomized Controlled Trials on Oral Chinese Herbal Medicine for Prostate Cancer

**DOI:** 10.1371/journal.pone.0160253

**Published:** 2016-08-04

**Authors:** Huijuan Cao, Yujie Mu, Xun Li, Yuyi Wang, Shiuan Chen, Jian-ping Liu

**Affiliations:** 1 Beijing University of Chinese Medicine, Beijing, China; 2 Beijing Aerospace General Hospital, Beijing, China; 3 Chongqing Traditional Chinese Hospital, Chongqing, China; 4 Department of Cancer Biology, Beckman Research Institute of the City of Hope, Duarte, California, United States of America; Carolina Urologic Research Center, UNITED STATES

## Abstract

**Background:**

Prostate cancer is the most common malignant tumor associated with male reproductive system.

**Objective:**

The existing eligible randomized controlled trials (RCTs) were critically appraised for the safety and effectiveness of CHM for prostate cancer.

**Methods:**

A literature search was conducted by using PubMed, CENTRAL, CNKI, CBM, VIP and Wanfang databases until August 2015. RCTs of CHM or CHM plus conventional medicine for prostate cancer patients were included. The primary outcomes appraised were survival time, time to progression and quality of life. The risk of bias assessment according to the Cochrane Handbook was used to evaluate the methodological quality of the included trials. Revman 5.3 software was used for data analyses. Risk ratio and mean difference (MD) with a 95% confidence interval (CI) were used as effect measures. Meta-analysis was to be used if sufficient trials without obvious clinical or statistical heterogeneity were available.

**Results:**

A total of 17 RCTs involving 1224 participants were analyzed. One trial was about CHM comparing to no treatment. The remaining 16 trials used CHMs as adjunctive treatment for endocrine therapy. Due to the poor quality of methodologies of most trials, only limited evidence showed that a combination of CHM and endocrine therapy might be more effective in restraining the development of the disease (MD 10.37 months, 95%CI 9.10 to 11.63 months), increasing patients’ survival time (7–15 months) or improving patients’ performance status, when compared to endocrine therapy alone (Karnofsky performance scale average changed 15 scores between groups). No severe adverse event was reported related to CHM.

**Conclusion:**

Due to the insufficient quality of trials that were analyzed, it is not appropriate to recommend any kind of CHMs in treating prostate cancer at the present time. Well-designed trials with high methodological quality are needed to validate the effect of CHMs for patients with prostate cancer.

## Background

Prostate cancer is the most common malignant tumor in the male reproductive system. Incidence of prostate cancer is known to relate to geographical area, ethnicity and age [1]. In Europe, USA and other developed countries, the mortality rate of the prostate cancer is only after lung cancer and colorectal cancer. This disease may cause serious harm to the physical and mental health of men as well as a heavy economic burden [2, 3]. The morbidity of prostate cancer in China and other Asian countries are currently low; however, changes of lifestyle and the improvement of living standards in recent years have caused an upward trend of this disease [4].

Radical resection of the prostate is an effective treatment for prostate cancer during early stage. But the applicability of surgery has to consider the patients’ age and the clinical stage of the cancer [5]. Because the test of the prostate specific antigen (PSA) is not very common in China, many patients are diagnosed at an advanced stage of the disease and thus have already missed the best treatment timing for surgery. Even the most commonly used endocrine therapy, maximal androgen blockade (MAB) by combining anti-androgen agents and surgical castration, was found associated with only a small survival benefit (about 5%) and increased adverse events[6, 7].

As a palliative treatment, traditional Chinese herbal medicine (CHM) was commonly used with oral administration for advanced prostate cancer in China. With a strong sense of the overview of a patient’s whole body, CHM does not just aim for tumor eradication, but focuses on improving the body’s strength and reducing the possibility of recurrence, as well as metastasis. Results from previous studies showed that CHM, as an adjunctive treatment following surgery, radiotherapy, chemotherapy, and endocrine therapy, may prolong survival time and improve the quality of life of prostate cancer patients [8]. Currently, CHM is considered more for its roles in treating advanced prostate cancer. However, systematic clinical research evidence for the effectiveness of CHM is still insufficient.

## Objectives

The aim of this review is to critically appraise the existing randomized controlled trials (RCTs) on CHM for treatment of prostate cancer, and provide evidence-based evaluation on the safety and effectiveness of oral administration of CHM against this cancer.

## Methods

The format of this review refers to the checklist of Preferred Reporting Items for Systematic Reviews and Meta-Analyses (see S1 Table).

### Inclusion criteria

RCTs which compared oral administration of CHM (alone or in combination with other treatments) to those without CHM in treating prostate cancer were included in our analysis. The primary outcomes included survival status (such as survival time and survival rate), quality of life, and time to progression (TTP); secondary outcomes included measurements of PSA, patient performance status, prostate volume (PV), serum testosterone, and adverse events. At least one of the above outcomes should be reported in the included trials in our evaluation.

### Search strategy

Two English databases and four Chinese databases were searched from inception to August 2015, including the Cochrane Central Register of Controlled Trials(CENTRAL), PubMED, China Network Knowledge Infrastructure(CNKI), Chinese Scientific Journals Database (VIP), Wanfang Database (for unpublished graduate theses in China), and Chinese Biomedicine (CBM). Details of the search strategies for PubMED were shown as below.

#1: Search "Medicine, Chinese Traditional"[Mesh]; #2: Search "Drugs, Chinese Herbal"[Mesh]; #3: Search "Prostatic Neoplasms"[Mesh]; #4: Search (#1 OR #2) AND #3

Above strategies were adopted for each specific database, and Chinese characters for relevant key words were used when searching Chinese databases.

### Study selection

Two authors (Cao H and Mu Y) independently screened the titles and abstracts of the achieved citations from primary searching. Full text of the articles of potential interest were download for further evaluation, those meeting inclusion criteria were included in the final review. Disagreements between the two authors were resolved by discussion and if needed, arbitrated by a third author (Liu JP).

### Data extraction and quality assessment

Two authors (Wang Y and Mu Y) extracted the data from the included trials independently on patient characteristics, intervention/control details, clinical outcomes and quality-related information. The discrepancies were resolved through consensus. Missing data were achieved through contacting with authors of the original studies.

Selection bias (random sequence generation and allocation concealment), performance bias (blinding of participants and personnel), detection bias (blinding of outcome assessment), attrition bias (incomplete outcome data), reporting bias (selective reporting), and other biases(determined according to sample size calculation method, inclusion/exclusion of criteria for patients' recruitment, comparability of baseline data, funding sources, and any other potential methodological flaw that might have influenced the overall assessment) were assessed according to the criteria from the Cochrane Handbook for Systematic Reviews of Intervention [9]. Three potential bias judgments: low risk, high risk, and unclear risk, were determined for each single trial during assessment. A judgment of low risk was made when all the seven items met the criteria as “low risk”, a judgment of high risk of bias was made when at least one of the seven items was assessed as “high risk”.

### Data analysis

Data were summarized using risk ratio (RR) with 95% confidence intervals (CI) for binary outcomes or mean difference (MD) with 95% CI for continuous outcomes. We used Revman 5.3 software from the Cochrane Collaboration for data analyses. Meta-analysis was used if the trials had an acceptable homogeneity on study design, participants, interventions, control, and outcome measures. Statistical heterogeneity was tested by examining I^2^ [10], meaning that an I^2^ larger than 50% indicated the possibility of statistical heterogeneity. Both fixed effect model and random effect model were used if there was possibility of statistical heterogeneity among trials. Publication bias was explored by funnel plot analysis. Subgroup analyses were conducted to determine the evidence for the different treatment duration if data were sufficient. Sensitivity analyses were used in order to determine whether the conclusions differed if eligibility was restricted to studies without high risk of bias; or if a fixed/random effect model had been applied.

## Results

### Results of the search

**Fig 1:**
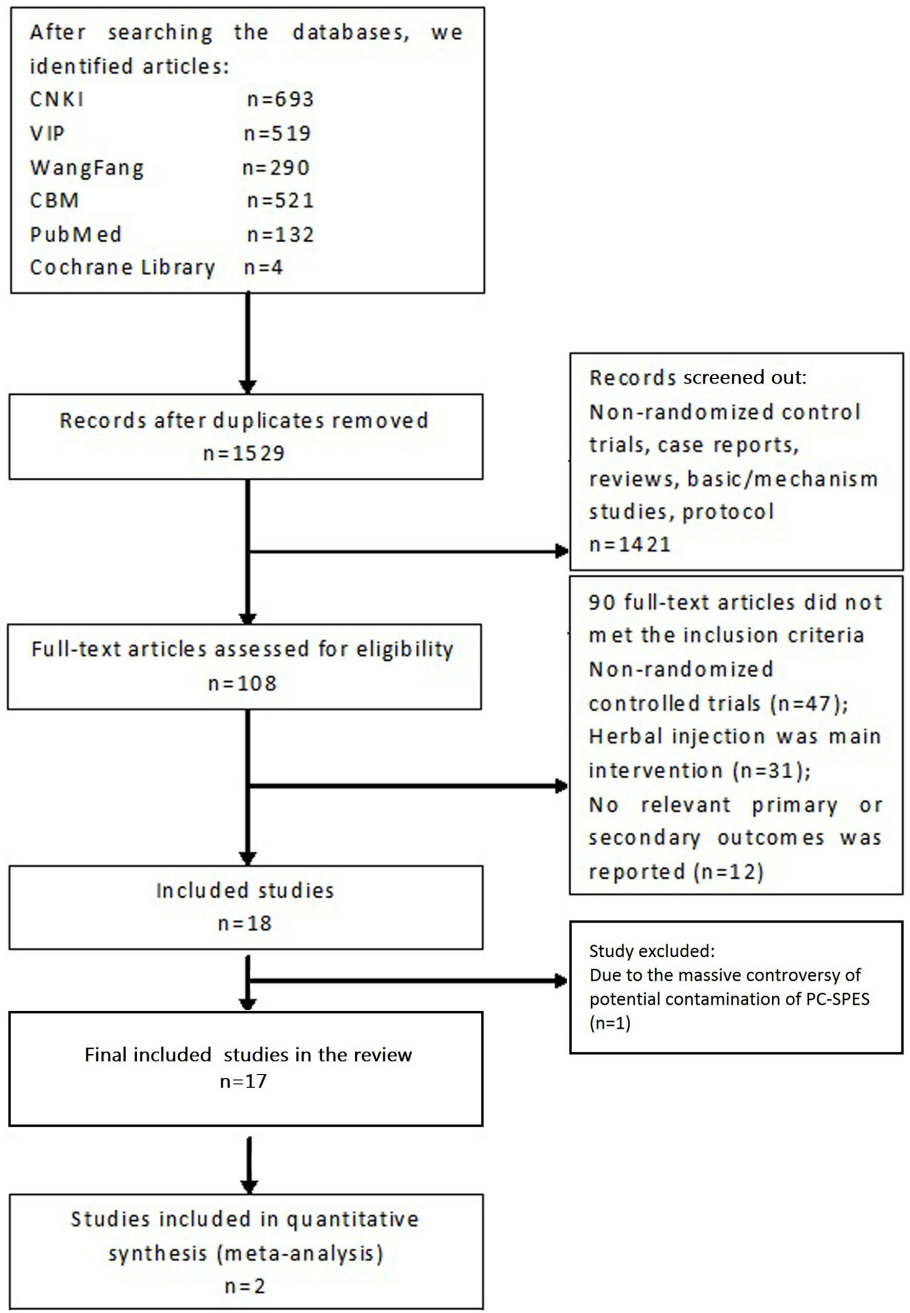
Study flow chart.

The search of 6 databases identified 1657 citations for further evaluation. Full texts of 109 articles were read, and 18 [11–28] trials met our inclusion criteria. However, one of the trial [28]tested a herbal capsule PC-SPES (960mg three times daily), which contains Ganoderma lucidum, Scutellaria baicalensis, Rabdosia rubescens, Isatis indigotica, Dendranthema morifolium, Seronoa repens, Panax pseudoginseng, and Glycyrrhiza uralensis. This herbal product was reported to be contaminated with diethylstilbestrol [29], thus the product may have a similar effect with diethylstilbestrol on inhibiting growth of both androgen-sensitive and androgen-independent prostate cancer cells. Due to the massive controversy of potential contamination of PC-SPES, the drug is no longer available in the U.S. Since it is hard to define whether it still belongs to herbal product, we excluded the trial from this review. Thus, finally 17 trials were included in the review. Details of the study flow were shown in Fig 1.

All of the 17 included trials were conducted in China and published in Chinese. Except for one four-arm trial [16], all the remaining trials were parallel two-arm trials. Characteristics of included trials were listed in Table 1.

**Table 1:** Characteristics of 17 included trials.

Study ID	Age (yrs)	Stage of the prostate cancer	Sample size(T/C)	Intervention	Control	Treatment Duration(months)	Outcomes	Follow up Duration
Chen 2013	65.8(54–80)	Not report	31/31	3 plus CHD (Yangyin Yishen Decoction) 150ml twice daily.	3	3	QOL (FACT-P); PSA	None
Dong 2007	T:68.2±3.2; C:68.0±3.1	*T: C-11 D-19; C: C-12 D-18	30/30	1 plus Chinese herbal pills (Juzao Pills) 10g three times daily	1	15	IPSS; QOL; PSA; PV	3.28±1.02 yrs
Gu 2007	T:68.3(57–83); C:70.1(57–82)	*T: C-5 D-11; C: C-7 D-7	16/14	3 plus CHM	3	6	PSA; QOL (QLQ-C30); Visual Analogue Scale; PV; SICM	6 months—3 yrs
Guo 2010	T: 74.2±4.0; C: 74.2±7.5	Not report	20/18	CHD (Chuanlong Yiai Decoction)	No treatment	3	PSA	None
Huang 2010	T: 70.3±2.5; C: 72.2±5.3	T_3-4_N_X_M_1C_-14; T_3-4_N_X_M_1b_-15	30/29	4 plus CHD (Body-strength and tumor depression Decoction) 100mL twice daily	4	3	Mortality; Survival time; TTP; SICM; KPS	Up to 8yrs
Huang 2014	T1: 73.1±3.2; T2: 72.7±3.1; C1: 73.1±2.8; C2: 72.8±2.6	T_3-4_N_X_M_1C_/T_3-4_N_X_M_1b_; T1:5/8 T2:7/6; C1:6/6 C2:5/7	T1: 13; T2:13; C1:12; C2:12	T1: 4 plus CHD (Body-strength and tumor depression Decoction) 100ml twice daily; T2: 3 plus CHD (Body-strength and tumor depression Decoction) 100ml twice daily	C1: 4; C2: 3	0.75	TTP; immune index; adverse events	7–48 (average 21.8) months
Jia 2013	T: 74.5; C: 75.5	*C -12 D_1_-29 D_2_-7	23/21	3 plus CHD (Yiqi Jiedu Quyu Decoction) 150ml twice daily	3	6	PSA; IPSS; QOL (QLQ-C30); SICM; Serum index; incidence of hot flash	None
Liu 2012	T: 68.0±2.3; C: 79.0±1.8	T_3-4_N_X_M_1C_/T_3-4_N_X_M_1b_; T:23/22; C:21/24	45/45	Chinese herbal granule (Jianpi Yishen Granule) 10g twice daily, based on control	Endocrine therapy	Not report	PSA; KPS; SICM; Immune index	None
Lu 2012	T:75.0±7.9; C:72.8±9.3	Not report	30/30	3 plus CHD 150mL twice daily	3	6	QOL; KPS; SICM; PSA; Serum index	None
Pang 2012	T: 65.7±8.6; C: 63.7±7.5	Not report	24/24	2 plus CHD (Qianlie Yuai Decoction) 150ml twice daily	2	3	PSA; KPS	None
Pang 2013	T: 72±6; C: 71±6	T:T_3_-10 T_4_-23; C:T_3_-11 T_4_-19	33/30	3 plus CHD (Qianlie Xiaozheng Decoction) 150ml twice daily	3	3	PSA; QOL (QLQ-C30); memorial anxiety scale for prostate cancer	None
Peng 2010	T: 71(61–79); C: 71 (59–79)	T: T_0_-15 T_1_-14 T_2_-51 T_3_-65 T_4_-3; N_0_-126 N_1_-3 N_2_-19; C: T_0_-12 T_1_-12 T_2_-57 T_3_-62 T_4_-4; N_0_-121 N_1_-5 N_2_-21	148/147	3 plus CHD twice daily	3	Not report	Survival time	4.7yrs
Tang 2014	T: 68.2; C: 67.4	T: III-11 IV-18; C: III-10 IV-20	29/30	3 plus CHD (Fuzheng Huayu Decoction) twice daily	3	3	KPS; PSA; immune index; adverse events	None
Wang 2009	T: 65.3±6.7; C: 67.4±8.3	T: T_1c_-4 T_2a_-5 T_2b_-4 T_t2c_-2; C: T_1c_-5 T_2a_-6 T_2b_-4 T_t2c_-2	15/17	2 plus CHD 100ml twice daily	2	T:6±2; C:6±3	PSA; Urination; Liver function	None
Zhang 2014	T: 67.8±8.1; C: 65.9±7.6	Not report	25/25	3 plus CHD (1^st^Qianliexianai Decoction) 125ml twice daily	3	3	PSA; QOL (FACT-P)	None
Zheng 2014	T: 66.4±9.2; C: 69.7±12.7	Not report	30/30	1 plus CHD (Jianpi Yishen Decoction) 150ml twice daily	1	3	SICM; Immune index; PSA; IPSS	None
Zhou 2013	T: 62.6±3.4; C: 65.3±2.9	Not report	42/42	3 plus CHD (Liuwei Dihuang Decoction) 150ml three times daily	3	Not report	PSA; Median survival time; Median TTP	None

*The stage of the disease was determined according to the Whitemore-Jewett criteria;

T: Treatment group; C: Control group; CHD: Chinese herbal decoction; QOL: quality of life; FACT-P: Functional assessment of cancer therapy-prostate; PSA: Prostate specific antigen;

IPSS: International Prostate Symptom Score; QLQ-C: Quality of life questionnaire-cancer; KPS: Karnofsky Performance Status; PV: Prostate volume; TTP: Time to progress;

SICM: Syndrome/Symptom integral of Chinese Medicine;

1: Castration (bilateral orchiectomy/triptorelin/leuprorelin/goserelin/estrogen);

2: Anti-androgen mono-therapy (megastrol/bicalutamide/flutamide);

3: Maximal androgen blockade (MAB): castration combined with anti-androgen (bicalutamide/flutamide);

4: MAB plus chemotherapy (docetaxel/prednison).

### Narrative of the included studies

Totally 1224 patients were included in this review, with an average of 72 patients per trial. Average age of those participants was 70 years old. The stages of the prostate cancer for majority of the patients were determined as III/IV, which mean the tumor has been infiltrated or beyond the prostate capsule.

CHM included herbal decoction (16 trials), herbal granule (1 trial), and herbal pill (1 trial). The herbal pill, called Juzao Pill (10g three times daily), which is composed of Flos chrysanthemi, Sargassum, Rhizoma sparganii, Semen strychni, Radix rhapontici, Pseudobulbus Cremastrae seu pleiones, Radix polygoni multiflori, Scolopendra, Flos lonicerae, Semen Irispallasii Fisch, and Pariphyllin, may strengthen body strength and soften solid masses, according to Chinese medicine theory. The herbal granule, Jianpi Yiqi Granule (10g twice daily), aiming to strengthen spleen and kidney of patients to alleviate the adverse events of chemotherapy and boost the immune system, was composed of Rhizoma atractylodis macrocephalae, Radix codonopsis, Fructus alpiniae oxyphyllae, Fructus lycii, Fructu sligustri lucidi, and Semen cuscutae. The 16 herbal decoctions (150ml twice daily) with 15 kinds of prescriptions, mainly work on strengthening body strength and suppressing the tumor growth. Details of the prescriptions of those 16 herbal decoctions were shown in Table 2.

**Table 2:** Components and adverse events of herbal prescriptions in 17 included trials.

Study ID	Herbal Medicine	Component of Prescription	Adverse events
Chen 2013	Yangyin Yishen Decoction	Astragalus mongholicus20g, Codonopsis pilosula20g, Rhizoma atractylodis macrocephalae15g, Radix ophiopogonis15g, Poria cocos10g, Radices rehmanniae15g, Turtle shell25g, Colla corii asini10g, Rhizoma polygonati15g, Herba hedyotis25g, Sculellaria barbata 25g, Semen coicis20g	Not reported
Dong 2007	Juzao Pills	Chrysanthemum, Rhizoma paridis, Curcuma zedoary, Pseudobulbus cremastrae seu pleiones, Sargassum, Gekko japonicas, Radix ginseng, Astragalus mongholicus, et al	Adverse event was not found in herbal medicine group
Gu 2007	Herbal decoction with tonifying Kidney-qi function	Rhizoma polygonati, Radix pseudostellariae, Radix morindae officinalis, Turtle shell, Lycium chinensis, Radix liquiritiae, Astragalus mongholicus, Pericarpium citri reticulatae, et al	Not reported
Guo 2010	Chuanlong Yiai Decoction	Rhizoma Chuanxiong, Ganodorma lucidum, Pberetima, Curcuma zedoary, Pseudo ginseng, Carthamus tinctorious, Herba taching	Adverse event was not found in herbal medicine group
Huang 2010	Body-strength and tumor depression Decoction	Astragalus mongholicus15g, Codonopsis pilosula20g, Rhizoma atractylodis macrocephalae 10g, Poria cocos 15g, Fructus aurantii 10g, Mangnolia officinalis 9g, Flos mume 10g, Sculellaria barbata20g, Polygonum cuspidatum 15g, Semen coicis 20g, Herba hedyotis 30g, Fructus amomi 10g,Fructus setariae germinatus 15g	Adverse event was not found in herbal medicine group
Huang 2014	Body-strength and tumor depression Decoction	Astragalus mongholicus15g, Codonopsis pilosula20g, Rhizoma atractylodis macrocephalae 10g, Poria cocos 15g, Fructus aurantii 10g, Mangnolia officinalis 9g, Flos mume 10g, Sculellaria barbata20g, Polygonum cuspidatum 15g, Semen coicis 20g, Herba hedyotis 30g, Fructus amomi 10g,Fructus setariae germinatus 15g	Adverse event was not found in herbal medicine group
Jia 2013	Yiqi Jiedu Quyu Decoction	Astragalus mongholicus30g, Radix pseudostellariae15g, Herba hedyotis 15g,Radix ranunculi ternati 15g,Fructus akebiae 15g, Prunella spike15g, Radix curcumae10g, Rhizoma curcumae longae10g, Herba plantaginis 15g,Cortex phellodendri 15g,Folium pyrrosiae 15g	Not reported
Liu 2012	Jianpi Yishen Granule	Codonopsis pilosula, Rhizoma atractylodis macrocephalae, Lycium chinensis, Fructus ligustri lucidi, Semen cuscutae, et al	Not reported
Lu 2012	Herbal decoction with conditioning spleen-stomach function	Codonopsis pilosula, Herba hedyotis, Rhizoma atractylodis macrocephalae, Radix liquiritiae, Poria cocos, Pericarpium citri reticulatae, Prunella spike, Pinellia ternata	Not reported
Pang 2012	Qianlie Xiaozheng Decoction	Semen coicis40g, Astragalus mongholicus15g, Rhizoma polygonati15g, Herba hedyotis15g, Rhizoma bolbostemmae 15g, Curcuma zedoary10g, Polyporus umbellatus10g	Not reported
Pang 2013	Herbal decoction with tonifying qi and yin function	Astragalus mongholicus, Radix ophiopogonis, Codonopsis pilosula, Radix puerariae, Fructus schisandrae, et al	Not reported
Peng 2010	Fuzheng Huayu Decoction	Astragalus mongholicus 30g, Radix pseudostellariae15g, Rhizoma atractylodis macrocephalae 15g, Poria cocos 15g, Radix paeoniae alba 15g, Radix paeoniae rubra 15g, Angelica sinensis 10g, Radix curcumae 10g, Radix achyranthis bidentatae 15g, Radix liquiritiae 6g	Not reported
Tang 2014	Adjusted Qianliexianai Decoction	Astragalus mongholicus, Angelica sinensis, Codonopsis pilosula, Poria cocos, Squama manitis, Duchesnea indica focke, Turtle shell, Gekko japonicas, Pseudobulbus cremastrae seu pleiones, chrysanthemum, Radix liquiritiae, et al	Symptoms of blood vessel constriction (9/29); post-castration syndrome (10/29); liver function damage(1/29);anemia (8/29)
Wang 2009	1st Qianliexianai Decoction	Radices rehmanniae15g, Rhizoma polygonati12g, Rhizoma atractylodis macrocephalae15g, Poria cocos15g, Semen coicis30g, Herba hedyotis30g, Rhizoma curcumae longae10g, Poligonum perfoliatum 30g	Not reported
Zhang 2014	Jianpi Yishen Decoction	Codonopsis pilosula30g, Rhizoma atractylodis macrocephalae12g, Poria cocos15g, Radix liquiritiae6g, Fructus ligustri lucidi15g, Semen cuscutae 15g, Fructus psoraleae 15g, Astragalus mongholicus30g, Turtle shell 15g, Herba hedyotis15g, Sculellaria barbata lOg	Not reported
Zheng 2014	Liuwei Dihuang Decoction	Radices rehmanniae, Rhizoma dioscoreae, Fructus corni, Cortex moutan, Poria cocos, Rhizoma alismatis, Curcuma zedoary, Rhizoma curcumae longae	Not reported
Zhou 2013	Yangyin Yishen Decoction	Astragalus mongholicus20g, Codonopsis pilosula20g, Rhizoma atractylodis macrocephalae15g, Radix ophiopogonis15g, Poria cocos10g, Radices rehmanniae15g, Turtle shell25g, Colla corii asini10g, Rhizoma polygonati15g, Herba hedyotis25g, Sculellaria barbata 25g, Semen coicis20g	Not reported

Types of comparisons included herbal decoction versus no treatment [14], herbal granule plus endocrine therapy versus endocrine therapy alone [18], herbal decoction plus endocrine therapy versus endocrine therapy alone, herbal decoction plus endocrine therapy and chemotherapy versus endocrine therapy and chemotherapy. Endocrine therapy included castration, anti-androgen mono-therapy, or maximal androgen blockade (MAB). Estrogen (Diethylstilbestrol 1mg three times daily, or estradiol valerate 1mg twice daily) as medical castration was used in 2 trials [12, 26]. Anti-androgen mono-therapy (megastrol 50mg twice daily, or flutamide 250mg three times daily) was involved in 2 trials [20, 24]. MAB, combined with castration (bilateral orchiectomy, or triptorelin/leuprorelin/goserrelin) and anti-androgen (bicalutamide 50mg once daily, or flutamide 250mg three times daily), was applied in 9 trials [11, 13, 17, 19, 21–23, 25, 27]. MAB combined with chemotherapy (docetaxel/prednison) was used in 2 trials [15, 16].

Primary evaluated outcomes of this review included survival status, Time to Progression (TTP), and Quality of Life (QOL). Survival time or survival rate was reported in 4 trials [12, 15, 22, 27], TTP or median TTP were reported in 3 trials [15, 16, 27], QOL (including QLQ-C30 (quality of life questionnaire for cancer patients), or Functional assessment of cancer therapy-prostate (FACT-P, including five sub-scales on assessing the physical status, social/family relationships, patients-practitioners relationship, emotional status, and body function)) were reported in 6trials [11–13, 17, 21, 25]. Secondary outcomes included PSA, performance status, international prostate symptom score (IPSS), PV and adverse events. PSA was reported in 14trials [11–14, 17–21, 23–27], Karnofsky performance scale (KPS) for evaluating the performance status was reported in 5 trials [15, 18–20, 23], IPSS was reported in 3 trials [12, 17, 26], and 3 trials [12, 13, 24] reported PV. Five trials mentioned adverse events [12, 14–16, 23]. Other outcomes measured in these 17 trials included Syndrome/symptom integral of Chinese Medicine, immune index, or serum index. Only 5 of the trials [12, 13, 15, 16, 22] mentioned follow-up duration, which varied from 6 months to 8 years.

### Methodology quality assessment

**Fig 2:**
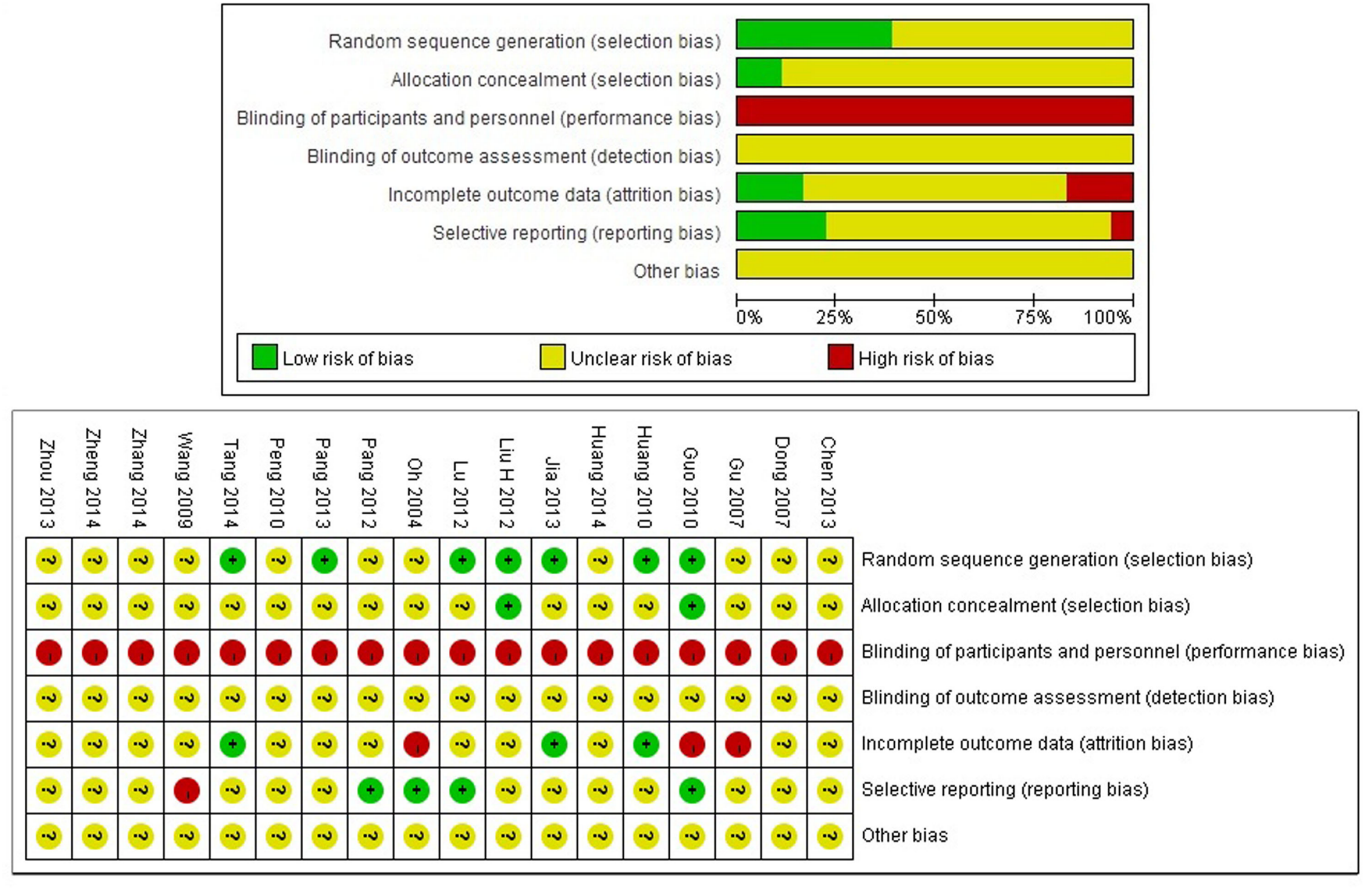
Summary of the Risk of bias assessment for each included trials.

According to the previously mentioned criteria, all included trials were assessed as having high risk of bias (Fig 2).

Only seven trials [14, 15, 17–19, 21, 23] reported the methods of randomization, all of them used a random number table, with one [15] employing statistical software to generate random numbers. Two among the seven trials [14, 18] used envelopes to contain the random numbers, and the remaining five trials did not mentioned whether or not they had adequate allocation concealment methods.

Sixteen of the included trials used CHM as add-on treatment for endocrine therapy (or combined with chemotherapy); however, placebo was not used in any of them. The remaining trial [14] compared CHM with no treatment without placebo control. Thus, all of the included trials were assessed as high risk of bias on the control factor of blinding to participants. Insufficient information was provided to judge the performance bias induced by lack of blinding to statistician and outcome assessors.

Only three included trials [15, 17, 23] were assessed as low risk of bias in incomplete outcome data, since they reported the numbers and reason for missing data in each group, and used adequate statistical methods (the last observation carried forward analysis) to deal with the missing data. Another two trials [13, 14] only analyzed the data from per-protocol set, and did not interpret results regarding the impact of missing data and reason for drop-out; thus, we evaluated these two trials as having high risk of attrition bias.

Protocols of thirteen of the included trials could not be found to assess the risk of reporting bias of the studies. Three[14, 19, 20] of the remaining four trials were assessed as low risk of reporting bias, and the other one [24] was evaluated as high risk of reporting bias due to the incomplete reporting of pre-defined outcomes.

No funding issue or methods of sample size calculation were apparent in all of the included trials, and it was difficult to determine whether a study was fraudulent. We evaluated all of the trials as at an unclear risk of other bias.

### Estimate effects of CHM for prostate cancer patients

#### Primary outcome measurements

*Survival time/rate*. Three trials [12, 15, 22] reported survival rates of participants with up to 8 years follow up duration. One trial [12] found no difference between CHM plus castration and castration alone on increasing survival rate (RR 1.08, 95%CI 0.86 to 1.36, 60 participants). The second trial [22] also showed no difference between CHM plus MAB and MAB alone on increasing survival rate (RR 1.04, 95%CI 0.92 to 1.18, 295 participants). The remaining trial [15] reported a small effect of CHM as an add-on treatment on increasing survival rate (RR 1.51, 95%CI 1.05 to 2.18, 59 participants), compared with MAB plus chemotherapy.

Two trials compared CHM plus MAB (and chemo-therapy) versus MAB (and chemo-therapy) alone and reported survival time of participants. However, one of them [27] did not report sufficient data for further analysis, which showed median survival time for CHM group was 25.2 months, and an obvious difference (p<0.01) was found between groups (18.9 months for MAB group). The other trial [15] showed significant difference between groups on this outcome (MD 15.87 months, 95%CI 11.90 to 19.84 months, 59 participants).

*Quality of life (QOL) assessment*. Six trials [11–13, 17, 22, 25] reported the quality of life through FACT-P (3 trials), QLQ-C30 (2 trials) or other relevant scales (1 trial). No meta-analysis was conducted due to the obvious clinical or statistical heterogeneity among trials.

All of the three trials [11, 21, 25] which mentioned the scores of FACT-P (pre and post treatment)in each subscale, showed that the herbal product (Yangyin Yishen Decoction, Qianlie Xiaozheng Decoction, and 1st Qianliexianai Decoction) as an adjunctive therapy had a better effect on improving patients’ quality of life compared with MAB alone (p<0.05). Similar results were found in two other trials [13, 17] for comparison of a combination of herbal medicine and MAB versus MAB alone when assessing patients’ quality of life through QLQ-C30 scale. 75% of patients in combination group (12 participants) got more than 6 scores changed after treatment, but in MAB group only 42.9% patients (6 participants) reported a similar improvement.

The remaining one trial [12] did not clarify which scales it employed for quality of life assessment; however, the result showed the combination of Yiqi Jiedu Quyu Decoction and MAB were better than MAB alone on improving patients’ quality of life (MD -16.00, 95%CI -19.11 to -12.89, 44 participants).

*The progress of the disease*. Only two trials reported time to progression (TTP, months) as an outcome measurement. One of them [16] was a four-arm trial which assessed the adjunctive effect of CHM for MAB or combination of MAB and chemo-therapy. Another trial [15] only compared CHM plus MAB and chemo-therapy versus MAB and chemo-therapy alone. The results showed a large effect of CHM as an add-on treatment for MAB (MD 6.60 months, 95%CI 5.50 to 7.70 months, 1 trial, 25 participants) or MAB plus chemo-therapy (MD 10.37 months, 95%CI 9.10 to 11.63 months, 2 trials, 84 participants, *I*^*2*^ = 0%, fixed-effect model) in restraining the development of the disease (including the radiographic progression and the progression to metastatic disease).

#### Secondary outcome measurements

*PSA*. Fourteen out of seventeen included trials reported PSA (ug/l or ng/ml) pre and post treatment; baseline PSA levels among trials were different and the treatment duration varied from 3 months to 15 months. Meta analysis was not conducted due to the obvious clinical and statistical heterogeneity among trials. One trial [12] showed a better effect of castration (prostatectomy, bilateral orcheictomy and diethylstilbestrol) than CHM (Juzao Pills) plus castration on reducing PSA (MD 4.13ug/l, 95%CI 2.37 to 5.89, 60 participants). Eight trials [11, 14, 18–21, 23, 25] showed a benefit of CHM for this outcome (at least 2.38 ug/l PSA lower in CHM group), and the remaining five trials [13, 17, 24, 26, 27] showed no difference between groups on controlling serum PSA in prostate cancer patients. Details of the results from each single trial were listed in Table 3.

**Table 3:** Estimate effect for Prostate Specific Androgen of the 17 included trials.

Study ID	Interventions	Sample size	Effect estimate (95%CI)	P value	
***Comparison 1*. *Herbal decoction versus no treatment***					
Guo 2010	ChuanlongYiai Decoction versus no treatment	40	MD -51.07 (-65.28 to -36.86)	<0.0001	
***Comparison 2*. *Herbal medicine plus endocrine therapy versus endocrine therapy***					
***2*.*1 Herbal medicine plus MAB versus MAB alone***					
Chen 2013	Yangyin Yishen Decoction plus MAB versus MAB alone	62	MD -7.10 (-9.59 to -4.61)		<0.00001
Gu 2007	Herbal decoction plus MAB versus MAB	30	MD -1.18 (-2.48 to 0.12)		0.08
Jia 2013	Yiqi Jiedu Quyu Decoction plus MAB versus MAB alone	44	MD -0.67 (-1.53 to 0.19)		0.13
Lu 2012	Herbal medicine plus MAB versus MAB alone	60	MD -55.65 (-67.87 to -43.43)		<0.00001
Pang 2013	Qianlie Xiaozheng Decoction plus MAB versus MAB alone	63	MD -24.60 (-46.58 to -2.62)		0.03
Tang 2014	Fuzheng Huayu Decoction plus MAB versus MAB alone	59	MD -2.38 (-3.30 to -1.46)		<0.00001
Zhang 2014	1^st^Qianliexianai Decoction plus MAB versus MAB alone	50	MD -5.20 (-8.78 to -1.62)		0.004
***2*.*2 Herbal medicine plus castration versus castration alone***					
Dong 2007	Juzao Pills combined with prostatectomy, bilateral orcheictomy and diethylstilbestrol versus castration alone	60	MD 4.13 (2.37 to 5.89)	<0.00001	
Zheng 2014	Jianpi Yishen Decoction plus progynova versus progynova alone	60	MD -4.59 (-10.01 to 0.83)	0.10	
***2*.*3 Herbal medicine plus anti-androgen mono-therapy versus anti-androgen mono-therapy alone***					
Pang 2012	Qianliexianai Decoction plus megestrol versus megestrol alone	48	MD -4.40 (-7.76 to -1.04)	0.01	
Wang 2009	Qianliexianai Decoction plus flutamide versus flutamide alone	33	MD -2.06 (-5.30 to 1.18)	0.21	
***2*.*4 Herbal medicine plus endocrine therapy (without details) versus endocrine therapy alone***					
Liu 2012	Jianpi Yishen Granule plus endocrine therapy versus endocrine therapy alone	90	MD -2.49 (-2.68 to -2.30)	<0.00001	

CI: Confidence Interval; MD: Mean Difference; RR: Risk Ratio; MAB: Maximal androgen blockade

*Performance status*. Within the five trials which reported KPS scores, three of them [15, 18, 19] showed a superior effect of herbal product as add-on therapy compared to MAB (MD 10.00, 95%CI 7.19 to 12.81, 60 participants), MAB plus chemotherapy (MD 19.12, 95%CI 15.27 to 22.97, 59 participants), or other endocrine therapy alone (MD 12.00, 95%CI 11.01 to 12.99, 90 participants) on improving patients’ performance status. One trial [23] found a similar number of patients whose KPS scores increased more than 10 between herbal medicine (Fuzheng Huayu Decoction) plus MAB group and MAB single applied group (RR 1.55, 95%CI 0.84 to 2.87, 25 participants). The remaining trial [20] only reported the KPS post treatment in both groups (52.79 in Qianlie Yuai Decoction plus control group, 50.54 in anti-androgen mono-therapy group) without the standard deviation or 95%CI of this outcome, thus we failed to interpret the data.

*Serum testosterone*. Only one trial [19] reported the change of serum testosterone after treatment, the result showed no difference between a combination of herbal decoction and MAB (Goserelin acetate sustained-release depot treatment plus Bicalutamide oral administration)and MAB alone for this outcome (MD -0.10 ng/ml, 95%CI -0.21 to 0.01, 60 participants) after six months of the treatment.

*Prostate Volume (PV)*. Two trials reported the patients’ PV before and after treatment; both of them showed that the average PV of the participants before the treatment was over 50 ml. However, one trial [12] found a significant benefit of herbal medicine (Juzao Pills) as add-on therapy for castration (Prostatectomy, bilateral orchiectomy and diethylstilbestrol) on reducing the volume of prostate (MD -5.20ml, 95%CI -5.67 ml to -4.73 ml, 60 participants), but the other trial [13] showed no difference between MAB therapies with or without herbal decoction administration (MD -2.61ml, 95%CI -6.62 ml to 1.40 ml, 30 participants) on PV after treatment.

*Adverse events*. Five trials mentioned adverse events in the herbal medicine group. Four of them [12, 14–16] reported there was no adverse event during the treatment duration. The remaining one trial [23] reported mild or moderate adverse events in Fuzheng Huayu Decoction groups. Symptoms of blood vessel constriction occurred in 31% of patients (9 cases), post-castration syndrome occurred in 34.5% of patients (10 cases), 27.6% of patients were reported to have anemia (8 cases), and 3.4% of patients had liver function damage (1 case) in combination of Fuzheng Huayu Decoction and MAB group. However, compared with control group (MAB), the combination group still showed lower incidence rate of adverse events (p<0.05). Details of reporting of adverse events were shown in Table 2.

#### Assessment for publication bias

Since none of the meta-analysis in this review included more than 10 studies, funnel plot was not conducted to assess the potential publication bias.

## Discussion

### Summary of findings

With concerns on 16 of the 17 trials, we have no confidence on determining the add-on effect of oral administration of herbal medicine for increasing survival rate and improving main symptoms (including PSA and PV) of patients with prostate cancer. Due to the poor methodological quality of the included trials and the insufficient number of trial participants, only limited evidence showed combination of herbal medicine and MAB may have better effect on increasing patients’ survival time (7–15 months) or improving patients’ performance status compared with MAB alone (KPS scores average changed 15 between groups).

The remaining trial [14] found an herbal decoction, Chuanlong Yiai Decoction, may have an effect on reducing PSA (MD -51.07ug/l) of patients with prostate cancer compared with no treatment. However, a conclusion could not be drawn due to the small sample size and potential high risk of bias in the trial.

Safety of herbal medicine was rarely reported in the included trials; thus, we could not make any confirmed conclusions on this outcome.

### Agreement and disagreement with other studies

We found one review [30] assessed the complementary and alternative medicine (CAM) for prostate cancer, in which herbal preparations were involved. However, only PC-SPES and Zyflamend (an anti-inflammatory proprietary blend of 10 standardized herbal extracts including turmeric, holy basil, green tea, huzhang, ginger, Chinese goldthread, oregano, Scutellaria baicalensis, and barberry) were mentioned in that review, and no data from a complete clinical trial could be used for evaluating those herbal preparations. The author demonstrated that the available data provided insufficient evidence to support the use of CAMs (including herbal preparations) for the treatment of prostate cancer patients.

Compared to the above review, our study included 17 trials with different types of herbal preparations. Though the strength of the evidence was low, we found potential effect of oral herbal preparations for patients with prostate cancer on some key symptoms’ improvement.

### Limitations of the review

Low levels of evidence in this review were mainly caused by the poor quality and small sample size of original included trials. The inconsistency of findings of herbal medicine’s effect on improving main symptoms of prostate cancer among these trials further reduced the internal validity of the evidence.

Although we searched both Chinese and English databases, all of the included trials were retrieved from Chinese literature, which may have introduced potential selection bias and limited the external generalization of the evidence.

Types of clinical stages of prostate cancer determine the details of treatment. In this review, almost half of the included trials did not report or classify clinical stages of the participants, which made the subgroup analysis regarding to the different clinical stages of the disease unavailable.

### Implications for the clinical practice

First aim of the treatment for patients with prostate cancer is to strengthen their self immune-function to alleviate the symptoms and get better quality of life. According to this review, herbs or herbal prescriptions with the function of strengthening the spleen and kidney “qi” are commonly used to achieve this objective. Top five of most frequently used herbs were Astragalus mongholicus (in 12 trials), Rhizoma atractylodis macrocephalae (in 9 trials), Codonopsis pilosula (in 9 trials), Herbah edyotis (in 9 trials), and Poriacocos (in 7 trials). The first three and Poriacocs were commonly used for tonifying (taking a tonic or nourishing food to build up) one’s qi and enhancing body immunity. Herba Hedyotis was reported to be used in several studies [31–33], which showed effects on enhancing specific or non-specific immunity and was recommended to be provided to cancer patients.

Herbal medicine may have effects on alleviating the symptoms or reducing the incidence rate of adverse events caused by endocrine therapy; however, insufficient evidence was seen supporting this conclusion.

### Implications for the future research

First of all, the protocol of the clinical trial should be pre-defined according to the research question. Ideally, preclinical data should be provided to support the design of the clinical trials. Authors are encouraged to register their study protocols before trial implementation to ensure the research can be conducted according to the pre-defined standard. In the protocol, the primary and secondary outcomes should be defined for the research. While one can argue whether PSA is a good marker for prostate cancer, it is the only approved marker to indicate the development of prostate cancer, and testing of serum PSA has been standardized. Therefore, PSA values should be included as a parameter to evaluate the outcome of prostate cancer trials. If possible, PSA doubling time during treatments should be determined. A documentation of a decrease of PSA could be more informative than a single measurement of PSA value. Secondly, there should be unified scales for assessing the quality of life of patients with prostate cancer (such as EORTC QLQ-PR25 and FACT-P). Therefore, those scales should be used and interpreted appropriately. Furthermore, in order to observe the long-term efficacy of treatment with assessment of patients’ survival time or time to progression, follow-up with adequate duration should be considered during clinical study.

According to this review, for outcomes like patients’ survival time or change of PSA after treatment, a combination of herbal medicine and endocrine therapy did not show advantages compared with endocrine therapies themselves. Thus, the sample size of future studies should be calculated carefully to make sure that data could achieve enough power for statistical analysis. Meanwhile, factors that may influence or downgrade the methodological quality of studies should be controlled during the research. Adequate methods are requested for random number generation, allocation concealment, missing data handling, avoiding performance bias and other bias. Reporting of the clinical study should follow the international standard or criteria, such as CONSORT statement [34].

In addition to treatment trials, prostate cancer prevention trials involving CHM should not be overlooked. Although it is not easy to carry out primary prevention trials, it is possible that the use of CHM could prevent or delay of the recurrence of prostate cancer. One of the authors (Shiuan Chen) has recently completed a trial to determine the effects of white button mushrooms (WBM) against biochemically recurrent prostate cancer [35]. Therapy with WBM appears to both impact PSA levels and modulate the biology of biochemically recurrent prostate cancer by decreasing immune-suppressive factors, such as myeloid-derived suppressor cells (MDSCs). This could be one way to evaluate anti-prostate cancer properties of CHM.

CHM has been applied in China for more than 2,000 years and shown to be useful in many ways. CHM is complex in nature and used mainly for symptom management. Our analysis was not able to reach a clear conclusion mainly due to inconsistency of the trial design, variable outcome measurements, and the complexity of CHM regimens. Furthermore, patient population might not be well controlled. It is also possible that during the trials, participants also took various supplements without telling the doctors. Through a careful and well-designed trial with a definitive outcome measurement, it is more likely that conclusions addressing the usage of CHM in prostate cancer treatment will be generated.

## Conclusions

Due to the insufficient quality of trials that were analyzed, it is not appropriate to recommend any kind of CHMs in treating prostate cancer at the present time. Well designed trials with high methodological quality are needed to validate the effect of CHMs for patients with prostate cancer.

## Supporting Information

S1 Table The PRISMA checklist. (DOC)

## References

[1] Brendler CB. Diagnosis and staging of prostate cancer. *Keio J Med*. 1988; 37: 10–23. 3286957 10.2302/kjm.37.10

[2] Woolam G. Cancer statistics, 2000: A benchmark for the new century. *CA Cancer Journal for Clinicians*.2000; 50: 7–33. 10.3322/canjclin.50.1.6 10735012

[3] Torre LA, Bray F, Siegel RL, Ferlay J, Lortet-Tieulent J, Jemal A. Global cancer statistics, 2012. *CA Cancer J Clin*. 2015; 65(2): 87–108. 10.3322/caac.21262 25651787

[4] Yang L, Yuan Y, Sun T, Li H, Wang N. Population-based cancer incidence analysis in Beijing, 2008–2012. *Chinese Journal of Cancer Research*.2015; 27(1): 13–21. 10.3978/j.issn.1000-9604.2015.01.07 25717221 PMC4329184

[5] Hu F. The analysis on the current status of epidemiology, diagnosis and treatment of prostate cancer in China. *Dissertation of Sichuan University*. 2006.

[6] Schmitt B, Bennett C, Seidenfeld J, Samson D, Wilt TJ. Maximal androgen blockade for advanced prostate cancer. *Cochrane Database of Systematic Reviews*. 1999; Issue 2. Art. No.: CD001526. 10.1002/14651858.CD001526 PMC10759791 10796804

[7] Heidenreich A, Bastian PJ, Bellmunt J, Bolla M, Joniau S, van der Kwast T, et al; European Association of Urology. EAU guidelines on prostate cancer. Part II: Treatment of advanced, relapsing, and castration-resistant prostate cancer. *Eur Urol*.2014; 65(2): 467–479. 10.1016/j.eururo.2013.11.002 24321502

[8] Peyromaure M, Debre B, Mao K. Management of prostate cancer in China: a multicenter report of 6 institutions. *The J of Urol*. 2005; 174: 1794–1797. 16217289 10.1097/01.ju.0000176817.46279.93

[9] Higgins J, Green S. Cochrane Handbook for Systematic Reviews of Interventions. Version 5.0.2 The Cochrane Collaboration 2009.

[10] Higgins JPT, Thompson SG, Quantifying heterogeneity in a meta-analysis. *Statist in Med*. 2002; 21(11): 1539–1558. 10.1002/sim.1186 12111919

[11] Chen ZF, Yu SL, Lin F. Influence of endocrine therapy combined with Yang Yin Yi Shen Tang on PSA value and life quality of advanced prostate cancer patients. *Jilin Journal of Medicine*. 2013; 34(22): 4450–4451.

[12] Dong SM. Clinical observation of integrative traditional Chinese and western medicine for 30 patients with prostate cancer. *Chinese Journal of Clinical Medicine Research*.2007; 13(4): 533–5.

[13] Gu ZM, Lv LG, Wang ZH, Chen ZQ. Clinical observation of integrative traditional Chinese and western medicine for prostate cancer. *Journal of Guangdong College of Pharmacy*.2007;(01):92–3.

[14] Guo WX. Clinical observation on the PSA of Chuan Long Yi Ai Decoction for Hormonal independent Prostate cancer. Dissertation for Master Degree of Guangdong University of Chinese Medicine. 2010.

[15] Huang GJ, Wang H, Chen CK, Li GH. Effect of Fuzheng Yiliu Decoction for survival time and quality of life of patients with late stage prostate cancer. *Jiangsu Journal of Traditional Chinese Medicine*. 2010; (06):18–20.

[16] Huang GQ, Li P, Song JL, Luo ZB, Guo L, Jiang G. Clinical study of body-strength and tumor-suppression decoction with chemotherapy resist later prostate cancer to local lesions development and metastasis after surgery of patients. *The Medical Forum*. 2014; 18(1): 1–4.

[17] Jia YJ, Li XJ, Li C, Zhao C. Clinical Efficacy Analysis of Treating Advanced Prostate Cancer by Yiqi Jiedu Quyu Recipe Combined Endocrine Therapy. *CJITWM*.2013; 33(4): 448–451. 23841259

[18] Liu H, Liu S, Wang H. Effect of Jianpi Yishen Granules for quality of life and immune function of prostate cancer patients after castration. *Chinese Journal of Information on TCM*.2012; 19(6): 61–2.

[19] Lu J. Summary of GaoRonglin’s experience on using conditioning spleen stomach methods for prostate cancer. *Dissertation for Doctor Degree of China Academy of Chinese Medical Science*.2012.

[20] Pang XS, Liu SJ. Effect of Qianliexianai Decoction for PSA and quality of life of patients with late-stage prostate cancer. *Information on Traditional Chinese Medicine*. 2012; (04): 50–2.

[21] Pang R, Lu JX, Gao XS, Liu B, Song SQ, Bo H. Clinical Study of Hormone-Refractory Prostate Cancer Treated by Qianliexiaozheng Decoction. *Journal of Surgery of Integrative Traditional Chinese and Western Medicine*.2013; 19(4): 374–377.

[22] Peng Y, Yao QB, Hu XH, Li X, Zhou GP. Combination of Yiqi Yangyin treatment and anti-androgen mono-therapy for patients with prostate cancer. *Chinese Journal of Andrology*. 2010; (06): 44–46, 53.

[23] Tang JW, Pei JW, He CH, Yang F. Clinical study of Fuzheng Huayu Decoction combined with endocrine therapy for later prostate cancer. *Forum on Traditional Chinese Medicine*.2014; 29(5): 15–16.

[24] Wang QH. Auxiliary endocrine therapy of prostate carcinoma of traditional Chinese medicine. Dissertation for Master Degree of Dalian Medical University. 2009.

[25] Zhang P, Lu ZJ, Su Y, Xu Y, Zhu QY, Gu XJ. Clinical observational study of integrative traditional Chinese and western medicine for 25 cases of prostate cancer. *Jiangsu Journal of Traditional Chinese Medicine*. 2014; 46(11): 39–40.

[26] Zheng WF. Castration resistant prostate cancer on the spleen and kidney part (Spleen kidney both deficiency type) in patients with the effect of life quality and immune function. Dissertation for Master Degree of Fujian University of Chinese Medicine. 2014.

[27] Zhou H, He XY, Zou QF. Effective Observation of Modified Liuwei Dihuang Decoction Combined with Bicalutamide on Treating Advanced Prostatic Cancer. *Journal of Sichuan of Traditional Chinese Medicine*. 2013; 31(6): 95–96.

[28] Oh WK, Kantoff PW, Weinberg V, Jones G, Rini BI, Derynck MK, et al Prospective, multicenter, randomized phase II trial of the herbal supplement, PC-SPES, and diethylstilbestrol in patients with androgen-independent prostate cancer. *J Clin Oncol*. 2004; 22(18): 3705–12 15289492 10.1200/JCO.2004.10.195

[29] Guns ES, Goldenberg SL, Brown PN. Mass spectral analysis of PC-SPES confirms the presence of diethylstilbestrol. *Can J Urol*. 2002; 9(6): 1684–1688. 12517310

[30] Klempner SJ, Bubley G. Complementary and alternative medicine in prostate cancer: from bench to bedside? *The Oncologist*. 2012; 17(6): 830–837. 10.1634/theoncologist.2012-0094 22618569 PMC3380882

[31] Wang YL, Zhang Y, Fang M, Li QJ, Jiang Q, Ming L. Immuno-mdulatory effects of total flavones of oldenlandia diffusa willd. *Chinese Pharmacological Bulletin*. 2005; 21(4): 444–447.

[32] Wang Q, Hou XQ, Cui XJ. Effect of Huangqin Baihuasheshecao Decoction on immune-status of patients with primary nephritic syndrome. *Shandong Journal of Medicine*. 2011; 51(9): 69–70.

[33] Chen YY. Pharmacology of Herba hedyotis. *Science and Technology Information*. 2007; (8): 194.

[34] Schulz K F, Altman DG, Mother D. CONSORT 2010 Statement: updated guidelines for reporting parallel group randomised trials.*Trials*.2010; 11:32 10.1186/1745-6215-11-32 20334632 PMC2857832

[35] Twardowski P, Kanaya N, Frankel P, Synold T, Ruel C, Pal SK, et al A phase I trial of mushroom powder in patients with biochemically recurrent prostate cancer: roles of cytokines and myeloid-derived suppressor cells (MDSCs) for *Agaricusbisporus* induced PSA responses, *Cancer*. 2015; e-pub ahead of print; 10.1002/cncr.29421 PMC5685188 25989179

